# Zinc-α2-Glycoprotein Modulates AKT-Dependent Insulin Signaling in Human Adipocytes by Activation of the PP2A Phosphatase

**DOI:** 10.1371/journal.pone.0129644

**Published:** 2015-06-11

**Authors:** Victòria Ceperuelo-Mallafré, Miriam Ejarque, Xavier Duran, Gisela Pachón, Ana Vázquez-Carballo, Kelly Roche, Catalina Núñez-Roa, Lourdes Garrido-Sánchez, Francisco J. Tinahones, Joan Vendrell, Sonia Fernández-Veledo

**Affiliations:** 1 Hospital Universitari de Tarragona Joan XXIII, Institut d´Investigació Sanitària Pere Virgili, Universitat Rovira i Virgili, Tarragona, Spain; 2 Centro de Investigación Biomédica en Red de Diabetes y Enfermedades Metabólicas Asociadas, Instituto de Salud Carlos III, Madrid, Spain; 3 Departament of Biochemistry and Molecular Biology II, School of Pharmacy, Complutense University, Instituto de Investigación Sanitaria del Hospital Clínico San Carlos, Madrid, Spain; 4 Hospital Universitario Virgen de la Victoria, Instituto de Investigaciones Biomédicas de Málaga, Málaga, Spain; 5 Centro de Investigación Biomédica en Red de Fisiopatología de la Obesidad y la Nutrición, Instituto de Salud Carlos III, Madrid, Spain; Tohoku University, JAPAN

## Abstract

**Objective:**

Evidence from mouse models suggests that zinc-α2-glycoprotein (ZAG) is a novel anti-obesity adipokine. In humans, however, data are controversial and its physiological role in adipose tissue (AT) remains unknown. Here we explored the molecular mechanisms by which ZAG regulates carbohydrate metabolism in human adipocytes.

**Methods:**

ZAG action on glucose uptake and insulin action was analyzed. β1 and β2-adrenoreceptor (AR) antagonists and siRNA targeting PP2A phosphatase were used to examine the mechanisms by which ZAG modulates insulin sensitivity. Plasma levels of ZAG were measured in a lean patient cohort stratified for HOMA-IR.

**Results:**

ZAG treatment increased basal glucose uptake, correlating with an increase in *GLUT* expression, but induced insulin resistance in adipocytes. Pretreatment of adipocytes with propranolol and a specific β1-AR antagonist demonstrated that ZAG effects on basal glucose uptake and GLUT4 expression are mediated via β1-AR, whereas inhibition of insulin action is dependent on β2-AR activation. ZAG treatment correlated with an increase in PP2A activity. Silencing of the PP2A catalytic subunit abrogated the negative effect of ZAG on insulin-stimulated AKT phosphorylation and glucose uptake but not on GLUT4 expression and basal glucose uptake. ZAG circulating levels were unchanged in a lean patient cohort stratified for HOMA-IR. Neither glucose nor insulin was associated with plasma ZAG.

**Conclusions:**

ZAG inhibits insulin-induced glucose uptake in human adipocytes by impairing insulin signaling at the level of AKT in a β2-AR- and PP2A-dependent manner.

## Introduction

Adipose tissue (AT), the largest energy reserve in the body, is now acknowledged as a major endocrine organ with important functions in carbohydrate and lipid metabolism. The functional integrity of AT in terms of glucose uptake is crucial for regulating intermediate metabolism. Transport of glucose across the plasma membrane is a fundamental mechanism not only to provide cells with basic requirements for energy yielding processes, but is also vital for clearing glucose from blood into tissues, a process normally stimulated by insulin. Insulin-stimulated glucose uptake in AT and skeletal muscle involves the recruitment of the insulin-sensitive transporter GLUT4 to the plasma membrane from an intracellular compartment, in a process mediated through the activation of the AKT/AS160 pathway [1]. Dysfunctional fat mass (e.g in obesity, cachexia and lipodystrophy) has profound effects on whole-body energy homeostasis and is a major risk factor for insulin resistance, dyslipidemia, type 2 diabetes and cardiovascular disease [2]. Zinc-α2-glycoprotein (ZAG), a soluble protein initially characterized as a lipid mobilizing factor upregulated in mice with cancer-related cachexia, is recognized as an adipokine with a potentially vital role in the control of adiposity, and therefore overall metabolic health [3]. Genetic studies point to ZAG as a putative candidate gene for body weight regulation since ZAG-knockout mice are susceptible to weight gain [4], whereas transgenic mice overexpressing ZAG exhibit weight loss [5]. Moreover, a beneficial effect of ZAG administration for reducing body weight, by favoring lipid mobilization and utilization, has been demonstrated in mice [6–8]. Emerging evidence suggests that ZAG expression in AT is inversely associated with body fat mass. Accordingly, ZAG gene expression is increased in cachectic mice with a profound loss of body fat [9] and also in subcutaneous AT of obese women on a low-calorie diet [10]. Conversely, ZAG expression is downregulated in obesity, both in mice [5, 11] and humans [12, 13], and an inverse association between AT ZAG expression and parameters of insulin resistance has also been described [11, 13–16]. Despite this knowledge, the clinical relevance of ZAG as a modulator of AT metabolism is unknown. A protective role for ZAG in maintaining appropriate fat mass and insulin sensitivity has been proposed, however, data on human serum levels of ZAG in relation to fat mass are controversial [5, 12, 13, 16, 17]. Similarly, conflicting results have emerged regarding circulating ZAG levels and insulin resistance indices. ZAG has been described as an adipokine with antidiabetic properties [8, 14, 18, 19], while other studies have failed to demonstrate a link between circulating ZAG levels and insulin resistance [12, 16], or have described a positive correlation between serum ZAG and insulin resistance [20] and fasting glucose [17]. Given this controversy, we have investigated the role and molecular mechanisms by which ZAG regulates insulin sensitivity in peripheral tissues, principally in adipocytes. We demonstrate that ZAG inhibits insulin-induced glucose uptake in human adipocytes by impairing insulin signaling at the level of AKT, in a PP2A-dependent manner. Though prospective cohort studies suggest that reduced ZAG expression in AT may be linked to the pathogenesis of insulin resistance, our data establish this adipokine as a negative modulator of insulin sensitivity.

## Materials and Methods

### Materials

Insulin, BSA, anti-β-actin antibody and propranolol hydrochloride were from Sigma-Aldrich (St. Louis, MO, USA). Zinc-Alpha-2-Glycoprotein (ZAG) Human HEK293 recombinant protein was purchased from Biovendor (Laboratory Medicine Inc., Palackeho, Czech Republic). The β1-adrenoceptor antagonist 1-[2-((3-Carbamoyl-4-hydroxy)phenoxy)ethylamino]-3-[4-(1-methyl-4-trifluoromethyl-2-imidazolyl)phenoxy]-2-propanol dihydrochloride (CGP-20712A) was from Tocris Bioscience (Bristol, UK). Culture media and sera were from Invitrogen (Paisley, UK). 2-Deoxy-D-[1-3H] glucose (11.0 Ci/mmol) was from PerkinElmer Life Sciences (Boston, MA, USA). Antibodies against phosphorylated (p)-IRS-1 (Tyr612), total IRS-1 and PP2A were from Millipore (Bedford, MA, USA). Antibodies against p-AKT1 (Ser473), total AKT, p-AS160 (Thr642) and total AS160 were from Cell Signaling Technology (Beverly, MA, USA).

### In vitro cell culture

The Simpson-Golabi-Behmel Syndrome (SGBS) preadipocyte cell line was kindly provided by Dr. Wabitsch (University of Ulm, Germany) and was used as a cellular model of human subcutaneous adipocytes [21]. LiSa-2 cells, kindly provided by Dr. Möller (University of Ulm, Germany), were used as a cellular model of visceral human adipocytes [22]. The human myogenic cell line LHCN-M2 was kindly provided by Dr. Woodring E. Wright (UT Southwestern Medical Center, USA) and used as a cellular model of human myoblasts [23]. PAZ6 was used as a representative model for human brown pre-adipocytes [24]. The HepG2 human liver cell line was obtained from the American Type Culture Collection (ATCC). Cells were differentiated as described [21, 22] and cultured in a standard humidified incubator (21% O_2_ /5% CO_2_). Before treatments, cells were incubated overnight in serum-free DMEM-low glucose (1000 mg/l). Cell pellets were lysed in RIPA buffer containing a Protease Inhibitor Cocktail (Sigma-Aldrich) and protein concentration was determined with the BCA Protein Assay kit (Pierce, Rockford, IL, USA).

### Gene expression analysis

Total RNA was extracted from adipose tissue/cells using the RNeasy Lipid Tissue Midi Kit (Qiagen, Hilden, Germany). Total RNA quantity was measured at 260nm and purity was assessed by the OD260/OD280 ratio. One microgram of RNA was retrotranscribed with random primers using the Reverse Transcription System (Applied Biosystems, Foster City, CA, USA). Quantitative gene expression was evaluated by real-time PCR (qPCR) on a 7900HT Fast Real-Time PCR System using the TaqMan Gene Expression Assay (Applied Biosystems). The following genes were evaluated: ADRB1 (Hs 02330048_s1), ADRB2 (Hs 00240532_s1), ADRB3 (Hs 00609046_m1), GLUT1 (Hs 00892681_m1), GLUT3 (Hs 00359840_m1) and GLUT4 (Hs 00168966_m1). Results were calculated using the comparative Ct method (2-ΔΔCt), and expressed relative to the expression of the housekeeping genes cyclophilin 1A (PPIA) (Hs 04194521_s1) and 18S (Hs 03928985).

### Glucose transport

After treatments, cells were stimulated for 30 min with insulin, and glucose uptake was measured during the last 10 min of culture by incorporation of 2-deoxy-D[1–3H]-glucose as described [21]. Glucose uptake rates were calculated as picomoles glucose taken up per 10 min per milligram protein, and results were expressed as the percentage of stimulation over basal (control = 100).

### Western blot analysis

Equal amounts of protein were separated on SDS-PAGE gels, then transferred to Immobilon membranes and blocked [21]. Immunoreactive bands were visualized using SuperSignal West Femto chemiluminescent substrate (Pierce) and images were captured using the VersaDoc imaging system and Quantity One software (Bio-Rad, Hercules, CA, USA).

### Transient transfection with small interfering RNA (siRNA)

siRNAs directed to human PP2A catalytic subunit (PP2A-Cα) and control (scrambled) siRNAs were purchased from Dharmacon (Lafayette, CO, USA). Human differentiated adipocytes were transfected with 100 nM of siRNA using Dharmafect 1 reagent as described [25]. At 48h post transfection, adipocytes were treated with ZAG as described in the respective figures. Cell lysates were collected and insulin sensitivity on glucose uptake and AKT phosphorylation status were measured. PP2A-Cα protein expression and PP2A phosphatase activity were assessed to evaluate the effectiveness of siRNA silencing.

### PP2A phosphatase activity

PP2A activity was determined in human adipocytes using the Ser/Thr PPase Assay (Promega Corp., Madison, WI, USA). Cell lysates were obtained with a phosphatase lysis buffer (20 mM HEPES, pH 7.4, 10% (vol/vol) glycerol, 0.1% (vol/vol) NP-40, 30 mM β-mercaptoethanol, 1 mM EGTA), and activity was measured using a PP2A-specific reaction buffer (50 mM imidazole, pH 7.2, 0.2 mM EGTA, 0.03% β-mercaptoethanol, 0.1 mg/ml BSA). Free phosphate generated from a phospho-peptide was quantified by measuring the absorbance of a molybdate-malachite green-phosphate complex at 600 nm [25].

### Clinical analysis

Subjects were recruited by the endocrinology and surgery departments at the University Hospital Virgen de la Victoria (Málaga, Spain) in accordance with the Helsinki Declaration (2008). The study protocol was approved by the “Virgen de la Victoria Ethics and Clinical Assay Committee” and written informed consent was obtained from all participants. All patients had fasted overnight before collection of blood samples. Samples were collected from subjects according to stratification by age, gender and body mass index (BMI). Subjects were classified by their homeostasis model assessment of insulin resistance index (HOMA-IR) as insulin-sensitive (HOMA-IR<2), low insulin-resistant (2<HOMAIR<4) or high insulin-resistant (HOMA-IR>4), as previously described [21]. All subjects were Caucasian and reported that their body weight had been stable for at least 3 months prior to the study. They had no systemic disease and all had been free of infection in the previous month before the study. Primary liver disease, cardiovascular disease, arthritis, acute inflammatory disease, infectious disease, neoplastic and renal diseases were specifically excluded by biochemical evaluation. Levels of plasma glucose were determined in an ADVIA1200 (Siemens AG, Munich, Germany) autoanalyzer using standard enzyme methods. Plasma insulin was determined by RIA (Coat-A-Count insulin; Diagnostic Products Corp., Los Angeles, CA). Sensitivity was 2.6 mU/ml, and intra- and interassay coefficients of variation (CVs) were less than 5%. Plasma ZAG levels were measured by sandwich ELISA (Biovendor Laboratory Medicine). The assay sensitivity was 0.673 ng/ml, and the intra- and interassay CVs were less than 5 and 6.6%, respectively [13, 26].

### Statistical analyses

Statistical analysis was performed with the Statistical Package for the Social Sciences software version 15 (SPSS). Experimental results are presented as mean±SEM from 3–4 independent experiments performed at least in duplicate. Statistical significance was tested with unpaired Student’s *t* test or one-way ANOVA followed by the protected least-significant different test. For clinical and anthropometrical variables, normal distributed data are expressed as mean value±SD. Differences in clinical variables, laboratory parameters, or expression variables between groups were compared using ANOVA with *post hoc* Scheffe. Interactions between factors as well as the effects of covariates and covariate interactions with factors were assessed by Pearson’s correlation analysis and General Linear Model Univariate Analysis. Correction for confounding and interacting variables was performed using stepwise multiple linear regression analysis.

## Results

### Zinc-α2-glycoprotein *per se* increases glucose uptake but impairs insulin action

**Fig 1:**
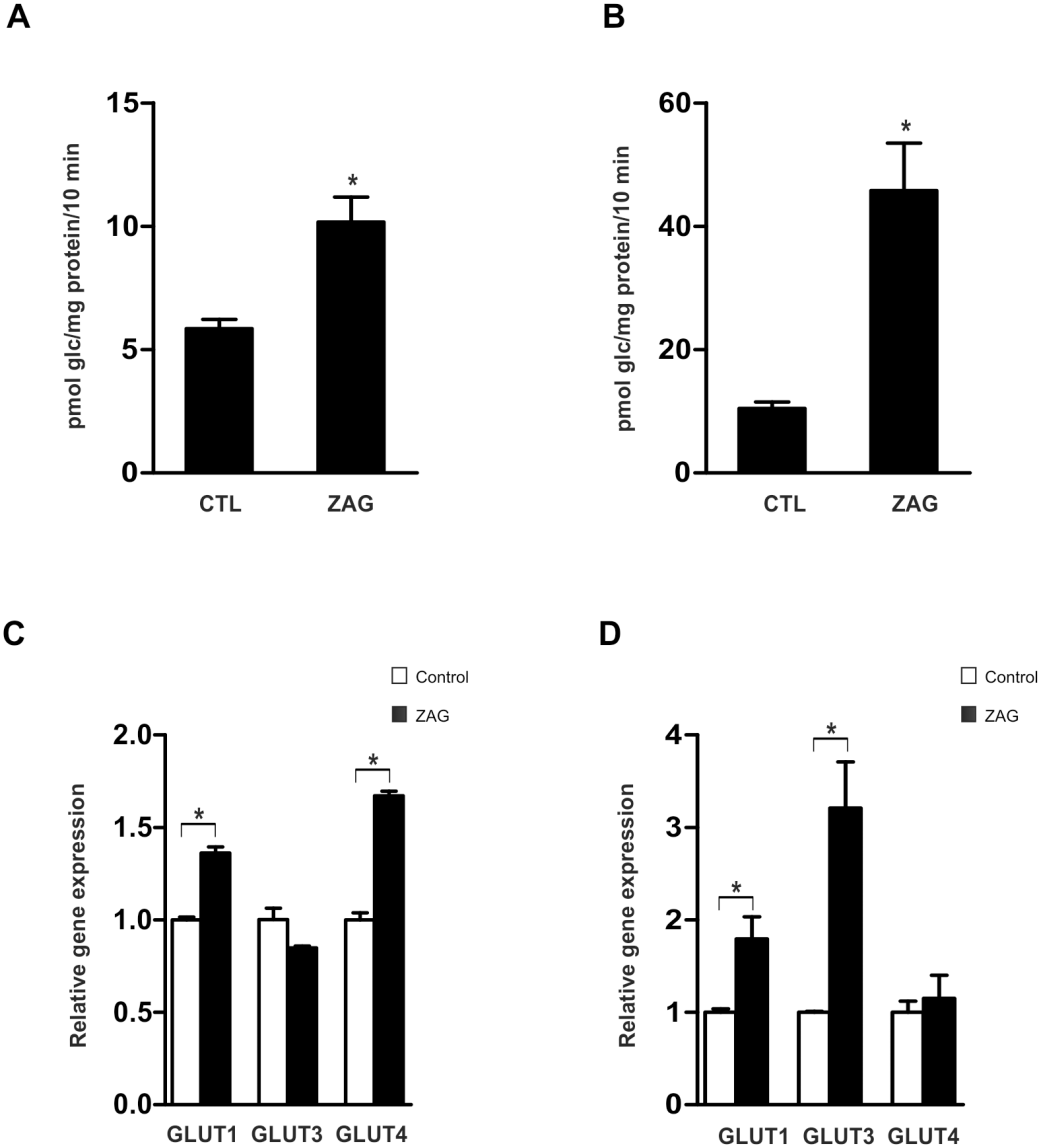
Administration of ZAG increases basal glucose uptake. Differentiated SGBS adipocytes **(A)** and LiSa-2 adipocytes **(B)** were cultured for 24 hours with or without 25 μg/ml ZAG. Glucose uptake was measured during the final 10 min by incorporation of labelled 2-deoxyglucose into the cells. Results are the mean±SEM of 3–4 independent experiments performed in triplicate. **(C)** Gene expression of GLUT1, GLUT3 and GLUT4 in SGBS and **(D)** LiSa-2 adipocytes treated or not with ZAG was analyzed by quantitative real time PCR (qPCR). Data are presented as mean±SEM (n = 3). *, P < 0.01 vs control.

**Fig 2:**
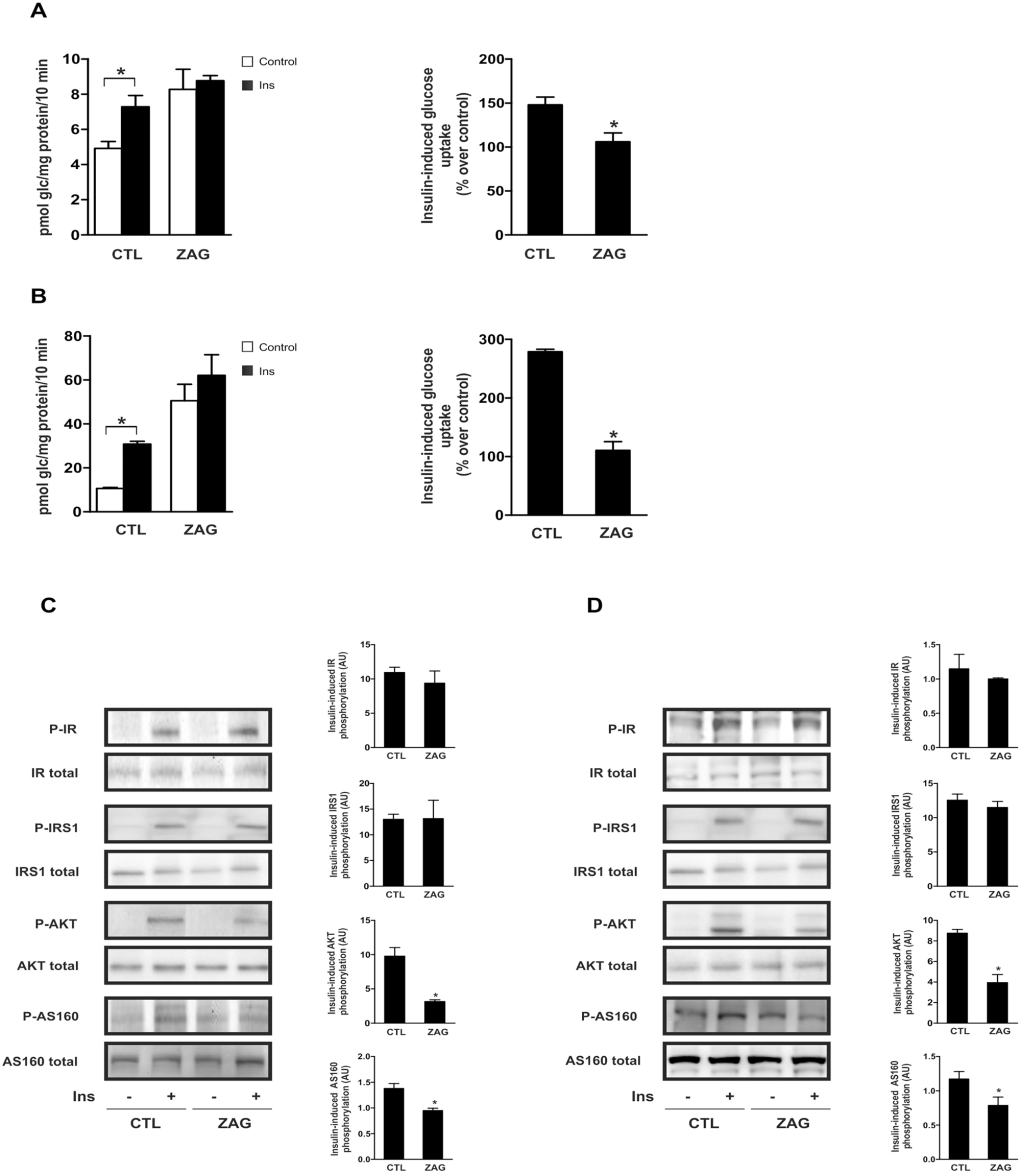
ZAG treatment impairs insulin action on glucose uptake and insulin signaling. Differentiated human subcutaneous SGBS adipocytes **(A)** and visceral LiSa-2 adipocytes **(B)** were cultured for 24 hours with or without 25 μg/ml ZAG before stimulation with 100 nM insulin (Ins) for 30 minutes. Glucose uptake was measured during the final 10 min by incorporation of labelled 2-deoxyglucose into the cells. Left panels represent mean±SEM of 3–4 independent experiments performed in triplicate and are expressed as pmol glc/mg prot/10 min. Right panels represent percentage of stimulation produced by insulin over control cells (no insulin, without or with ZAG respectively). *, P < 0.01. **(C)** Lysates from differentiated SGBS cells and **(D)** LiSa-2 cells cultured with or without 25 μg/ml ZAG for 24 hours before stimulation with 100 nM insulin (Ins) for 15 minutes, were analyzed by western blotting using antibodies against phosphorylated and total IRβ (Tyr1150/1151), IRS1 (Tyr612), Akt (Ser473) and AS160 (Thr642). A representative experiment is shown together with densitometric analysis of phosphorylated *vs* total proteins (3 independent experiments). *, P < 0.01.

We first explored the impact of ZAG on glucose uptake in human adipose cell lines previously described as robust models of differentiated adipocytes. Considering the maximal stimulatory effect on lipolysis described in previous *in vitro* experiments [7], cells were treated with 25 μg/ml ZAG for 24 h and basal glucose uptake rate was calculated. Compared with control conditions, administration of ZAG increased basal glucose uptake in human subcutaneous (Fig 1A) and visceral (Fig 1B) adipocytes. This was paralleled by a significant increase in mRNA expression of glucose transporters GLUT1 and GLUT4 in SGBS mature adipocytes (Fig 1C), and GLUT1 and GLUT3 in LiSa-2 cells (Fig 1D). Given this, we investigated whether ZAG exposure affected insulin-stimulated glucose uptake. Interestingly, insulin treatment of ZAG-exposed human adipocytes failed to further increase glucose uptake in subcutaneous (Fig 2A) and visceral adipocytes (Fig 2B). Comparable results were obtained in a cellular model of human mature brown adipocytes (S1A Fig) and also myocytes (S1B Fig). Thus, although single administration of insulin and ZAG activated glucose uptake, the effect was not additive. Accordingly, when insulin stimulation on glucose uptake was expressed as a percentage over basal levels, ZAG administration abolished insulin stimulation in all cells analyzed (Fig 2A and 2B and S1A–S1B Fig, right panels). Insulin resistance occurs when normal circulating concentrations of the hormone are insufficient to regulate carbohydrate and lipid metabolism. Thus, by definition, insulin resistance is a defect in signal transduction [27]. To determine whether the insulin-signaling cascade was affected by ZAG, we used western blotting to measure expression of key insulin-related proteins. Insulin stimulation of insulin receptor (IR) and insulin receptor substrate (IRS)1 tyrosine phosphorylation was not affected by ZAG treatment (25 μg/ml, 24 h), neither in mature human SGBS (Fig 2C) nor in LiSa-2 adipocytes (Fig 2D). In contrast, ZAG treatment significantly impaired insulin-induced AKT phosphorylation (Fig 2C and 2D). More importantly, insulin-stimulated phosphorylation of the AKT substrate AS160, which controls GLUT4 intracellular retention and release to the cell surface [1], was also significantly impaired in cells treated with ZAG (Fig 2C and 2D). Similar results on AKT phosphorylation were observed in other insulin-sensitive tissues such as hepatocytes (S2 Fig). Notably, neither basal glucose uptake and GLUT mRNA expression nor insulin action was affected by short-term treatment (3–6 h) with ZAG (data not shown). Thus, in a manner similar to other cytokines [28] and pathological situations associated with obesity-related insulin resistance, such as hypoxia [21] or hyperinsulinemia [22], ZAG produces paradoxical effects on glucose uptake in human adipocytes: it increases glucose uptake but also impairs insulin signaling, affecting insulin-stimulated glucose uptake.

### Zinc-α2-glycoprotein is a β1- and β2-adrenoreceptor agonist in human white adipocytes

**Fig 3:**
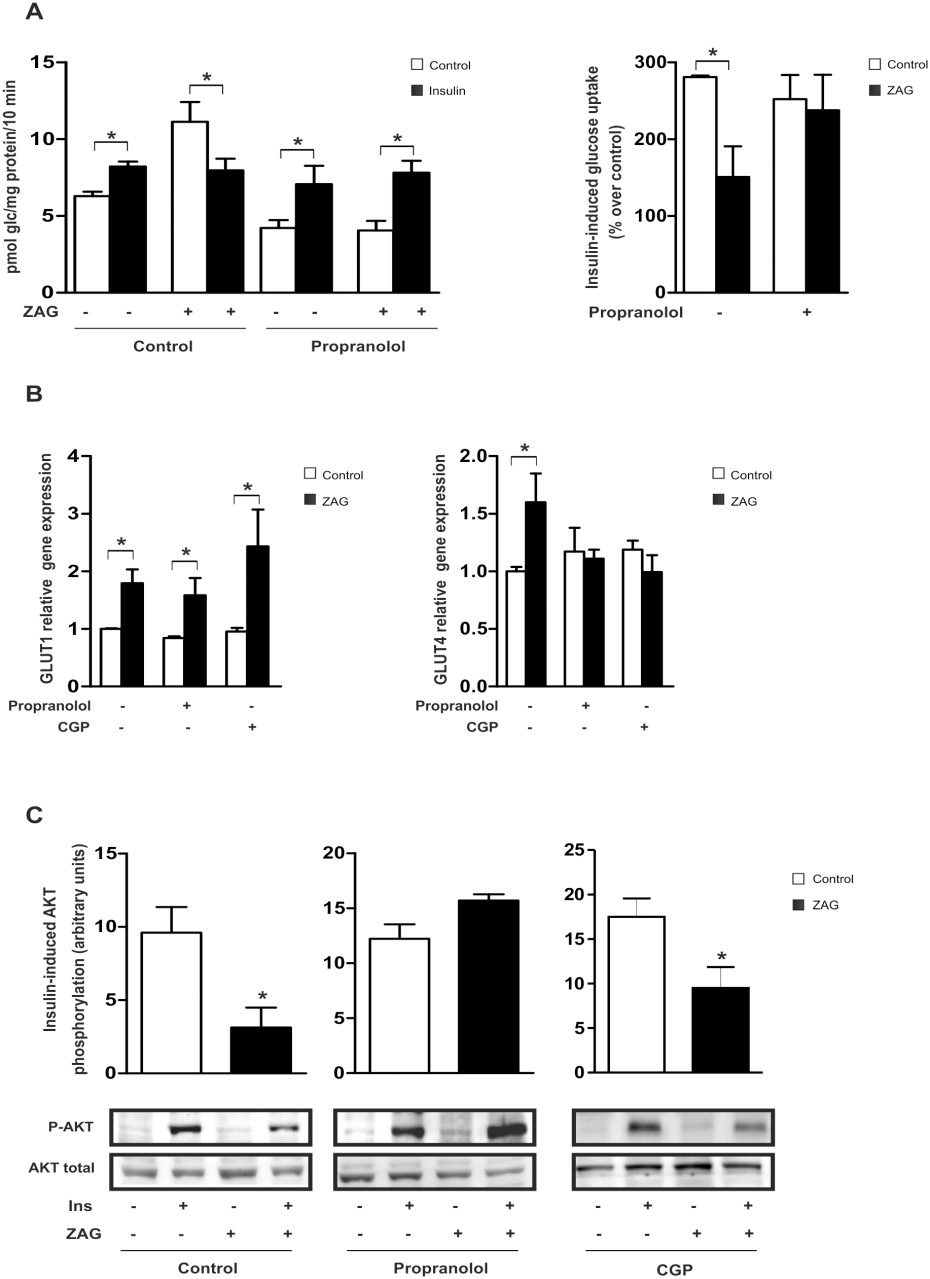
ZAG inhibits insulin action *via* β1/β2-AR signaling. Differentiated SGBS adipocytes were pre-treated for 30 min with or without 1 μM propranolol prior to culture with 25 μg/ml ZAG for 24 hours. **(A)** Glucose uptake was measured after stimulation with 100 nM insulin (Ins) for 30 minutes by incorporation of labelled 2-deoxyglucose into the cells for the final 10 minutes of culture. Left panels represent mean±SEM of 3 independent experiments performed in triplicate and are expressed as pmol glc/mg prot/10 min. Right panels represent percentage of stimulation produced by insulin over control cells (no insulin, without or with ZAG respectively). *, P < 0.01. **(B)** GLUT1 and GLUT4 mRNA expression were analyzed by qPCR in adipocytes pre-treated with 1 μM propranolol or 300 nM CPG20712A (CPG) prior to culture with ZAG. Data are presented as mean±SEM (n = 3). *, P < 0.01 vs control. **(C)** Lysates from differentiated SGBS cells pre-treated with propranolol or CPG prior to culture with ZAG and 100 nM insulin (Ins) for 15 minutes were analyzed by western blotting using antibodies against phosphorylated and total Akt (Ser473). A representative experiment is shown together with densitometric analysis of phosphorylated *vs* total proteins (3 independent experiments). *, P < 0.01.

The metabolic effects of ZAG in murine models have been primarily associated with β3-AR activation [7, 29]. It is widely accepted, however, that β3-AR expression in human adipose tissues is extremely low [30]. Indeed, a survey of β-AR mRNA expression in mature SGBS adipocytes demonstrated that β1-AR and β2-AR account for 65% of total β-AR expression (S3 Fig). To further explore the molecular mechanisms by which ZAG modulates glucose uptake and insulin sensitivity, mature SGBS adipocytes were pre-treated with the mixed β1/β2-AR antagonist, propranolol, prior to ZAG treatment and insulin stimulation. Propranolol treatment decreased ZAG-induced basal glucose uptake (Fig 3A, left panel), which correlated with the abolishment of ZAG-mediated effects on GLUT4 but not GLUT1 mRNA expression (Fig 3B). Furthermore, compared with control cells that did not receive propranolol, ZAG-mediated resistance to insulin-stimulated glucose uptake was abrogated in cells pretreated with the β1/ β2-AR antagonist (Fig 3A, right panel). Consequently, whereas ZAG treatment reduced the levels of phosphorylated AKT, pretreatment with propranolol restored insulin-induced activation of AKT in human adipose cells (Fig 3C). To question the potential role of β1-AR in regulating ZAG-mediated effects, SGBS mature adipocytes were treated with the specific β1-AR antagonist CGP20712A [31] prior to ZAG and/or insulin treatment. Pre-treatment with the β1-AR antagonist inhibited ZAG-mediated induction of GLUT4 mRNA expression (Fig 3B); however, insulin signaling was not reestablished (Fig 3C). Collectively, these results indicate that the effects of ZAG in this cell type might be specifically dependent on β1/β2-AR signaling, and suggest that the β3-AR subtype does not play a significant role in the insulin resistant state in adipose cells. Moreover, whereas the effects of ZAG on basal glucose uptake and GLUT4 expression are mediated via β1-AR, inhibition of insulin signaling seems to be dependent on β2-AR activation.

### Zinc-α2-glycoprotein decreases insulin sensitivity in human white adipocytes by a mechanism dependent on PP2A activation

**Fig 4:**
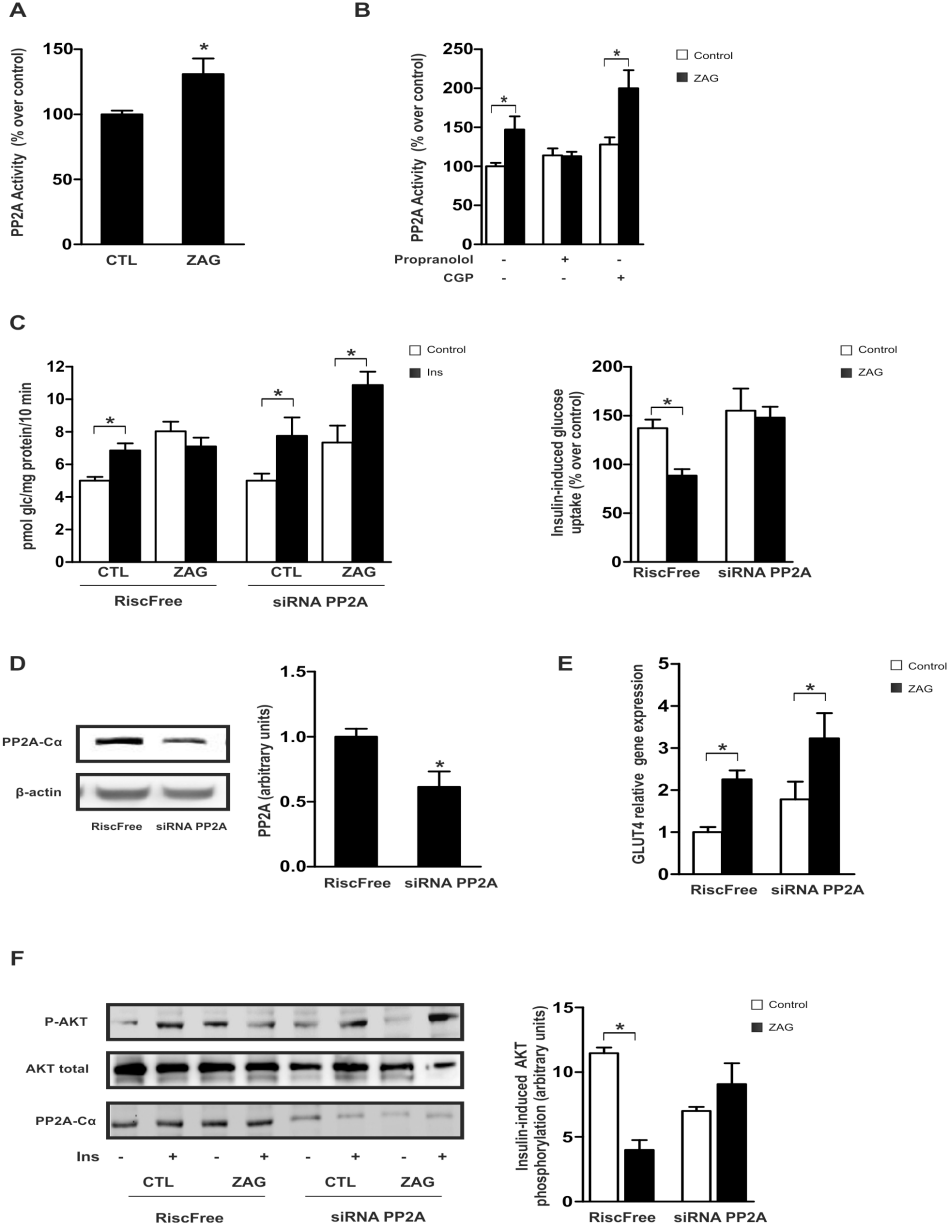
ZAG activation of PP2A phosphatase impairs insulin-stimulated glucose uptake and AKT phosphorylation in SGBS adipocytes. **(A)** Differentiated human adipose cells were cultured with 25 μg/ml ZAG for 24 hours and PP2A activity was measured. **(B)** PP2A activity was measured in mature adipocytes pre-treated with 1 μM propranolol or 300 nM CPG20712A (CPG) prior to culture with ZAG. Data are presented as mean±SEM (n = 3). *, P < 0.01 vs control. (**C**) Adipose cells were transfected with 100 nM siRNA against the α-catalytic subunit of PP2A (PP2A-Cα) or RISC-free (control cells) and cultured with or without 25 μg/ml ZAG for 24 hours prior to stimulation with 100 nM insulin (Ins) for 30 minutes. Glucose uptake was measured by incorporation of labelled 2-deoxyglucose into the cells during the last 10 minutes of culture. Results from 3 independent experiments performed in triplicate are expressed as pmol glc/mg prot/10 min. Right panels represent percentage of stimulation produced by insulin over control cells (no insulin, without or with ZAG, respectively). *, P < 0.01. **(D)** PP2A-Cα protein expression was analyzed by western blot in control and transfected cells. A representative experiment is shown together with densitometric analysis (3 independent experiments). *, P < 0.01. **(E)** GLUT4 mRNA levels were analyzed by qPCR in siRNA control and PP2A-Cα-silenced adipocytes in the absence or presence of ZAG. Data are presented as mean±SEM (n = 3). *, P < 0.01 vs control. **(F)** Cells transfected as in D were cultured or not with 25 μg/ml ZAG for 24 hours prior to stimulation with 100 nM insulin (Ins) for 15 minutes. Cell lysates were analyzed by western blotting using antibodies against phosphorylated and total Akt (Ser473). A representative experiment is shown together with densitometric analysis of phosphorylated *vs* total proteins (3 independent experiments). *, P < 0.01.

We hypothesized that the serine/ threonie phosphatase PP2A, a well-known negative regulator of insulin signaling by promoting de-phosphorylation and inactivation of AKT [32], could be regulated by ZAG in our cellular model. Treatment of SGBS (Fig 4A) or LiSa-2 adipocytes (S4 Fig) with ZAG for 24 h resulted in a significant increase in PP2A activity. To question whether ZAG-stimulated PP2A activation could be dependent on β2-AR signaling, adipocytes were pre-treated with a mixed β1/β2-AR antagonist and PP2A was measured following ZAG treatment. Results showed that pre-treatment with proranolol inhibited ZAG-induced PP2A activation (Fig 4B). In contrast, pre-treatment of adipocytes with a specific β1-AR antagonist (CGP20712A) did not affect PP2A activation in response to ZAG. To determine the potential role of PP2A in the insulin resistant state triggered by ZAG, PP2A gene expression was silenced with a specific siRNA duplex against the PP2Aα catalytic subunit (PP2A-Cα). Interestingly, in contrast to control-siRNA cells, ZAG-provoked resistance to insulin-stimulated glucose uptake was abrogated in PP2A-Cα-silenced adipocytes (Fig 4C). Western blot analysis confirmed the decrease in PP2A protein in silenced cells but not control-siRNA cells (Fig 4D). Of note, no effects were observed in basal glucose uptake (Fig 4D) or GLUT4 mRNA levels (Fig 4E) in PP2A-knockdown adipocytes treated with ZAG compared with control cells. More importantly, in line with its effects on insulin sensitivity for glucose uptake, the inhibitory effect of ZAG on insulin-stimulated AKT phosphorylation was abolished in PP2A-silenced cells compared with control-siRNA cells (Fig 4F). Collectively, these results strongly suggest that ZAG negatively affects insulin sensitivity on glucose uptake by reducing insulin-induced AKT activation, in part, through PP2A activation.

### Circulating levels of zinc-α2-glycoprotein are unchanged in a lean patient cohort stratified for HOMA-IR

Finally, given the controversy over circulating ZAG levels and insulin sensitivity in the clinical setting of obesity, plasma ZAG protein levels were measured in lean subjects (BMI ranging from 21.07 to 25.6) classified according to their insulin resistance, assessed by HOMA-IR. As shown in Table 1, no differences in circulating ZAG levels were detected between insulin-sensitive (HOMA-IR<2), low insulin-resistant (2<HOMAIR<4) and high insulin-resistant (HOMA-IR>4) subjects. Serum levels of ZAG were neither related to fasting plasma glucose nor baseline insulin as revealed by bivariate and multiple regression analysis (R = -0.37, p = 0.822 and R = 0.044, p = 0.788, respectively). Thus, despite the fact that some clinical studies show that ZAG is inversely correlated with BMI, glucose and insulin in a cohort including overweight patients (BMI range of 19.51–28.79) [19], our results in a lean cohort, not biased by BMI, suggest that ZAG might not be a useful biomarker of insulin sensitivity.

**Table 1:** Clinical, anthropometric, and analytical characteristics (units) according to insulin resistance classification.

	HOMA-IR<2	2<HOMA-IR<4	HOMA-IR>4
**N**	24	9	6
**Age (years)**	43 ± 9.175	50.11 ± 14.51	57.83 ± 16.62^a^
**Sex (male/female)**	12/12	3/6	3/3
**BMI (kg/m^2^)**	22.78 ± 1.71	23.46 ± 2.14	22.84 ± 1.02
**Insulin (μIU/ml)**	5.53 ± 1.80	10.49 ± 2.56 ^b^	22.02 ± 6.63 ^b^
**HOMA-IR**	1.28 ± 0.45	2.69 ± 0.68 ^b^	5.45 ± 1.86 ^b^
**DBP(mmHg)**	77.83 ± 12.96	79.33 ± 8.67	84.33 ± 14.98
**SBP (mmHg)**	117.50 ± 17.65	129.33 ± 23.24	131.67 ± 25.34
**Glucose (mg/dl)**	93.71± 11.05	104.00 ± 11.43	99.50 ± 8.19
**ZAG (mg/l)**	43.23 ± 6.48	42.98 ± 9.90	43.13 ± 5.60

The results are given as the mean±SD. BMI: body mass index; HOMA-IR: homeostasis model assessment of insulin resistance index; DBP: Diastolic Blood Pressure; SBP: Systolic Blood Pressure. ^a^ and ^b^ indicate significant differences between the means of the different groups:

^a^: P<0.05

^b^: P<0.01(ANOVA or Student´s t where appropriate).

## Discussion

ZAG, which was initially proposed as a tumour-derived cancer cachexia factor, is also produced by some normal tissues including AT [9]. Previous studies in rodents point to ZAG as an important player in modulating whole-body and AT insulin sensitivity [4–7, 18]. However, cross-sectional and interventional studies in humans have produced conflicting results regarding the link between circulating ZAG and insulin resistance [8, 12, 14, 16–20]. To date, the relevance of ZAG as a direct modulator of insulin sensitivity in human cells had not been explored. In the present study, we provide the first analysis of the metabolic effects of ZAG on carbohydrate metabolism with regards to insulin sensitivity and show that ZAG functions to inhibit insulin signaling. Thus, ZAG might act as a paracrine/autocrine regulator of human adipose cells, where it specifically stimulates lipolysis [29] but attenuates insulin signaling and, in consequence, insulin-induced glucose uptake, through a mechanism dependent on PP2A activation.

ZAG is abundantly secreted from mature adipocytes [11, 33], and ZAG gene expression in AT is inversely associated with body fat mass in both mice and humans in the context of obesity [11, 12] and also in cancer-induced cachexia [9, 11, 34]. Nevertheless, AT does not appear to significantly contribute to circulating ZAG, and AT-secreted ZAG seems to correlate more with nutritional status rather than fat mass, both in malignant and nonmalignant conditions [35]. In this scenario, ZAG is considered a catabolic factor in humans by virtue of its lipolysis-promoting activity [34]. Results from murine models postulated that ZAG could induce selective reduction in body fat, promoting lipolysis and increasing overall fatty acid oxidation [7]. Nonetheless, the effects of ZAG on UCP1 expression and energy expenditure in murine obesity models of obesity are far from clear [36].

**Fig 5:**
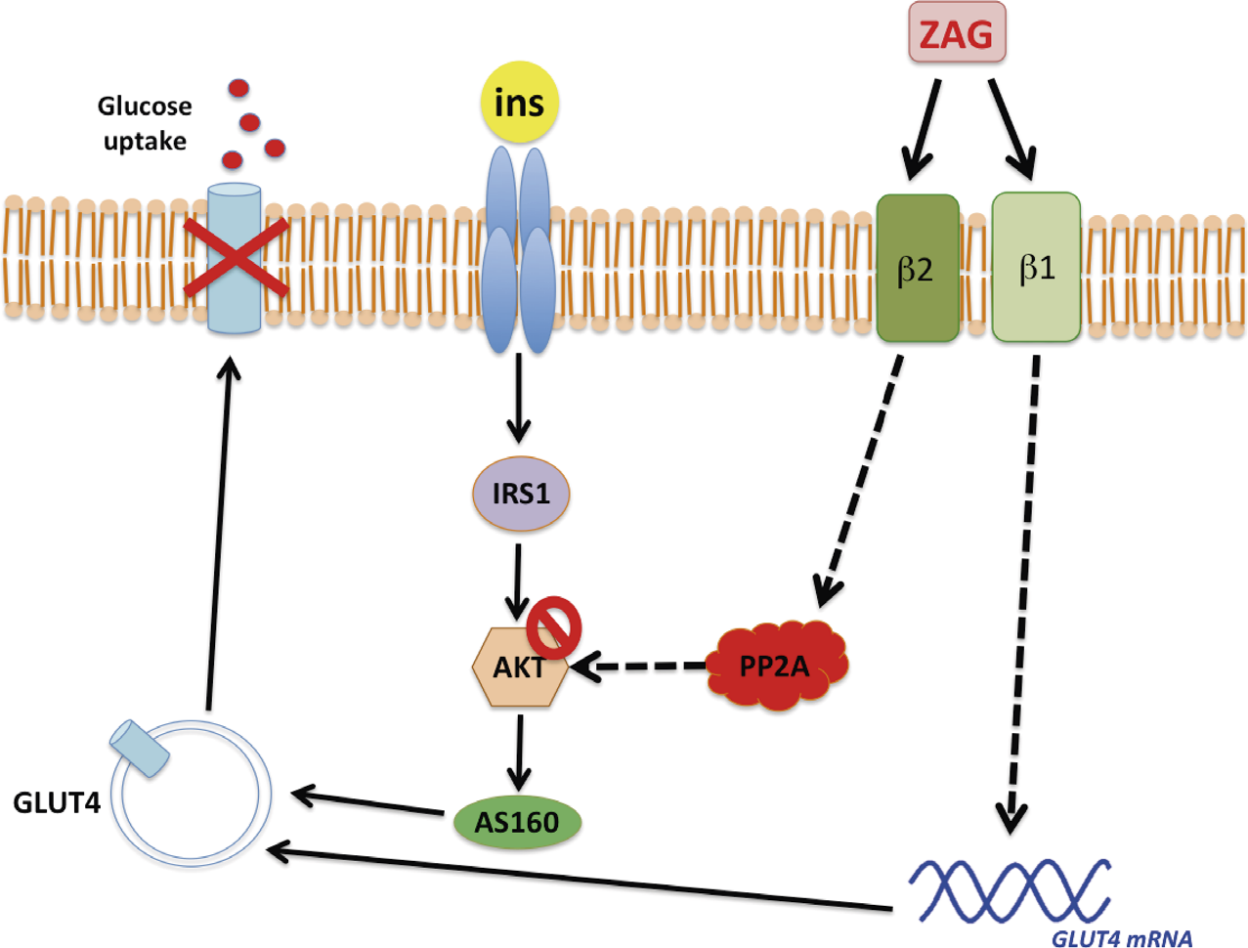
ZAG increases basal glucose uptake but impairs insulin-induced glucose uptake in human subcutaneous adipocytes by acting as a β1/2-AR agonist. ZAG enhanced GLUT4 gene exression and basal glucose uptake via β1-AR. In addition, ZAG might also activate PPA2 via β2-AR, inhibiting insulin-induced AKT phosphorylation and, in consequence, insulin-induced glucose uptake. Although some prospective cohort studies point to ZAG expression in AT as a useful biomarker to predict insulin sensitivity, our data establish ZAG as a negative modulator of insulin action.

Although controversial, it has been also suggested that ZAG might inversely reflect the status of insulin sensitivity in obesity. This notion is supported by the positive association found between the expression of ZAG in AT and some key components of the insulin signaling pathway, such as IRS1 [14] and GLUT4 [16]. However, results regarding circulating ZAG levels and insulin resistance indices such as HOMA-IR are conflicting [12, 16, 20]. Moreover, not only obesity but also cachexia and HIV-associated lipodystrophy, where ZAG expression in AT has been inversely associated to fat mass, are related with insulin resistant states [26]. Thus, some caution should be exercised when extrapolating information from observational clinical studies in pathological conditions in an attempt to understand the physiological function of a protein. Indeed, the inverse association between circulating ZAG levels and HOMA in obesity, found by some authors [19], was not observed in a cohort of lean patients classified according to HOMA-IR (this work). Our study clearly shows that treatment of human adipocytes with ZAG increases basal glucose uptake but induces an insulin resistant state, similar to other catabolic adipokines such as TNF-alpha [28]. Analogous to other stress conditions related to insulin resistance [21, 22], ZAG produces paradoxical effects in human subcutaneous adipocytes. Accordingly, ZAG increases glucose uptake, correlating with an increase in GLUT1 and GLUT4 expression, but also activates PP2A, inhibiting insulin-induced AKT phosphorylation and in turn, glucose uptake (Fig 5). This detrimental effect is also observed in other insulin sensitive cells such as myocytes and hepatocytes, suggesting a non-tissue-specific effect of ZAG. Although previous studies by Tisdale et al. have reported that the effects of ZAG on body weight and insulin sensitivity in ob/ob mice might be mediated through β3- or possibly β2-ARs [37], a recent report demonstrates that ZAG does not behave as a typical β3/2-AR agonist [36]. Our own results would suggest that ZAG elicits its inhibitory effects on insulin signaling in human fat cells by acting as a β2-AR agonist, whereas basal glucose responses are controlled by ZAG signaling through β1-AR (Fig 5). These findings are in agreement with the important metabolic differences described for rodent and human AT, where β3-AR would represent a minor player in human white AT [30]. It is well known that β-adrenergic agonists can stimulate glucose uptake in several tissues independently of insulin [38, 39]. Insulin and β2-adrenoreceptor pathways seem not to be additive and utilize similar mechanisms to increase glucose uptake, however, some differences have been revealed in specific insulin-signaling intermediates [39]. In agreement with the paradoxical effects observed with ZAG, other GPCR agonists distinct to those activating β-ARs, such as endothelin-1, stimulates glucose uptake but also desensitizes cells to acute insulin treatment [40]. Our study also provides compelling evidence for the participation of the Ser/Thr phosphatase PP2A as an effector molecule in the ZAG signaling cascade. PP2A has been primarily described as a negative regulator of the insulin signaling pathway since it impairs AKT activation [32]. Nevertheless, certain transduction pathways activated by some insulin-sensitizing adipokines are also elicited by PP2A activation [25, 41], suggesting that PP2A specificity depends on its regulatory subunits.

Based on the literature and the present results, we propose a model of regulation whereby ZAG might be a local factor activated in response to an energy-rich demand. Thus, the decreased production of ZAG in human obesity would be merely a reflection of increased fat mass and not directly related to insulin resistance. Moreover, considering that increased insulin-dependent glucose utilization is associated with lipogenesis, it is conceivable that ZAG, a well-established catabolic factor with a well-defined lipolytic effect, has negative effects over anabolic hormones such as insulin, which additionally show antilipolytic actions. Although a number of questions remain to be answered, overall, our study questions the use of ZAG as a biomarker of insulin sensitivity as suggested by some correlation data from clinical studies.

## Supporting Information

S1 Fig ZAG treatment provokes insulin resistance on glucose uptake in human sensitive cells. Differentiated human brown PAZ6 adipocytes **(A)** and LHCNM2 myocytes **(B)**, were cultured for 24 hours in the absence or presence of 25 ìg/ml ZAG, before stimulation with 100 nM insulin (Ins) for 30 minutes. Glucose uptake was measured during the final 10 min by incorporation of labelled 2-deoxyglucose into the cells. Results represent mean ± SE of 3–4 independent experiments performed in triplicate and are expressed as percentage of stimulation over non-treated cells (100%) (left panels) and as percentage of stimulation produced by insulin over control (right panels). *, P < 0.01.
(TIF)

S2 Fig ZAG treatment impaired insulin-stimulated AKT phosphorylation in human hepatocytes. HepG2 hepatocytes were treated cultured for 24 hours in the absence or presence of 25 ìg/ml ZAG, before stimulation with 100 nM insulin (Ins) for 15 minutes, and phosphorylated and total Akt (Ser473) was analyzed by western blotting. A representative experiment is shown together with densitometric analysis of phosphorylated vs total protein (3 independent experiments). *, P < 0.01.
(TIF)

S3 Fig ADRB mRNA expression in mature SGBS adipocytes. Expression of ADRB1, ADRB2 and ADRB3 mRNA was analyzed by qPCR in differentiated SGBS cells. Data are expressed as percentage of total. ADRB1: Adrenergic receptor, beta 1; ADRB2: Adrenergic receptor, beta 2; ADRB3: Adrenergic receptor, beta 3.
(TIF)

S4 Fig ZAG significantly increases PP2A activity. Differentiated LiSa-2 cells were incubated with 25ìg/ml ZAG for 24 hours, and PP2A activity was measured as described in Materials and Methods. Results represent means ± SE of 3–4 independent experiments. Significant differences: *, p<0.01 vs. control.
(TIF)
