# Supplementary figures and images for: Zinc-α2-Glycoprotein Modulates AKT-Dependent Insulin Signaling in Human Adipocytes by Activation of the PP2A Phosphatase
Source: PLoS One. 2015 Jun 11;10(6):e0129644. doi: 10.1371/journal.pone.0129644 (PMC4465909; doi:10.1371/journal.pone.0129644)

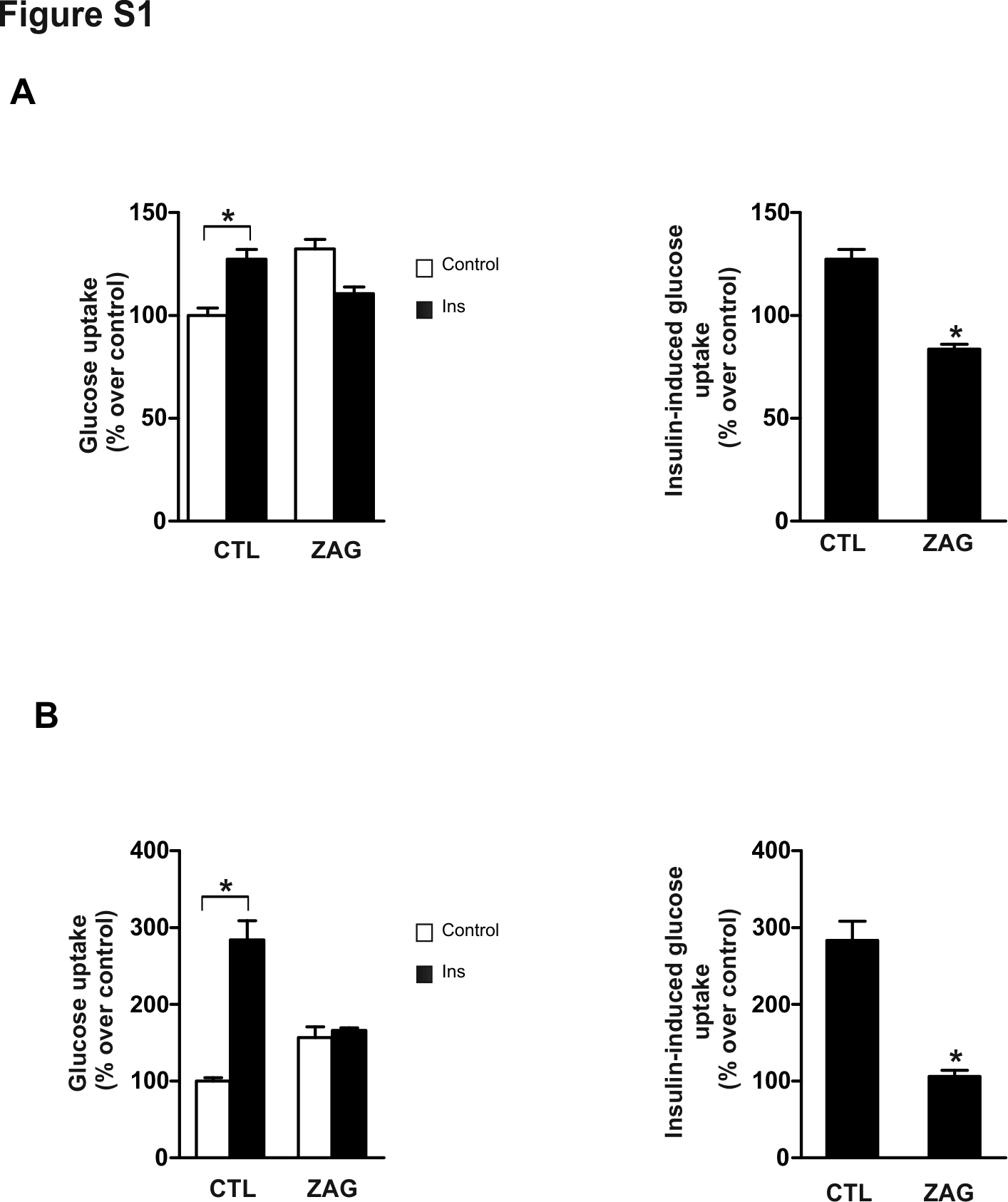

Supplement: S1 Fig — Differentiated human brown PAZ6 adipocytes (A) and LHCNM2 myocytes (B), were cultured for 24 hours in the absence or presence of 25 ìg/ml ZAG, before stimulation with 100 nM insulin (Ins) for 30 minutes. Glucose uptake was measured during the final 10 min by incorporation of labelled 2-deoxyglucose into the cells. Results represent mean ± SE of 3–4 independent experiments performed in triplicate and are expressed as percentage of stimulation over non-treated cells (100%) (left panels) and as percentage of stimulation produced by insulin over control (right panels). *, P < 0.01. (TIF) [file pone.0129644.s001.tif]

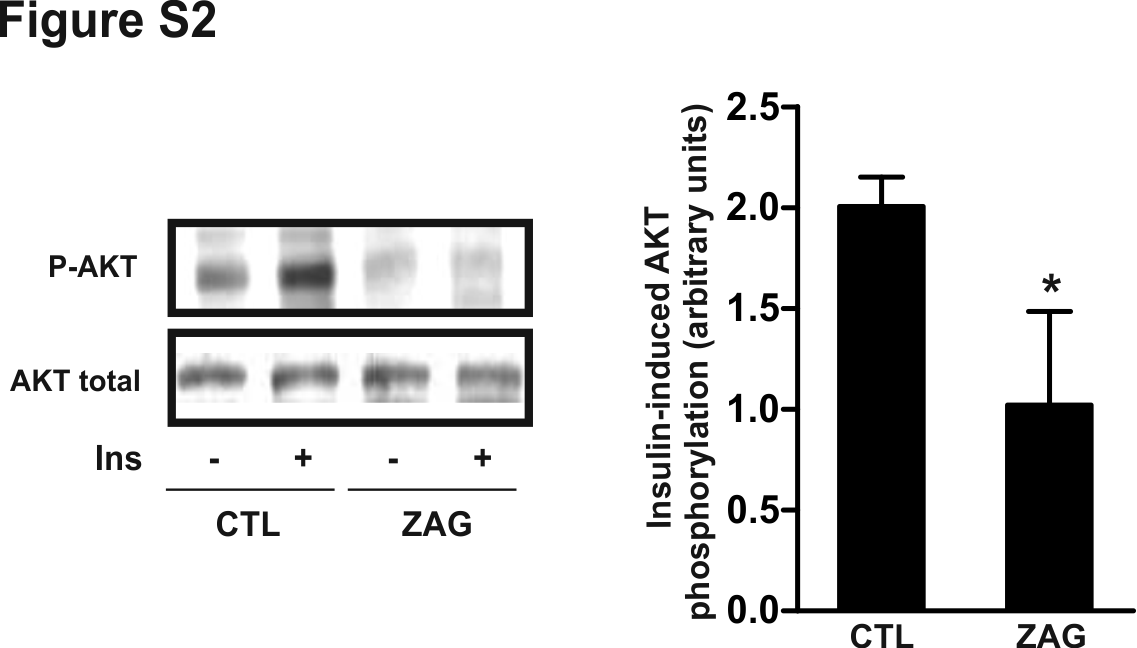

Supplement: S2 Fig — HepG2 hepatocytes were treated cultured for 24 hours in the absence or presence of 25 ìg/ml ZAG, before stimulation with 100 nM insulin (Ins) for 15 minutes, and phosphorylated and total Akt (Ser473) was analyzed by western blotting. A representative experiment is shown together with densitometric analysis of phosphorylated vs total protein (3 independent experiments). *, P < 0.01. (TIF) [file pone.0129644.s002.tif]

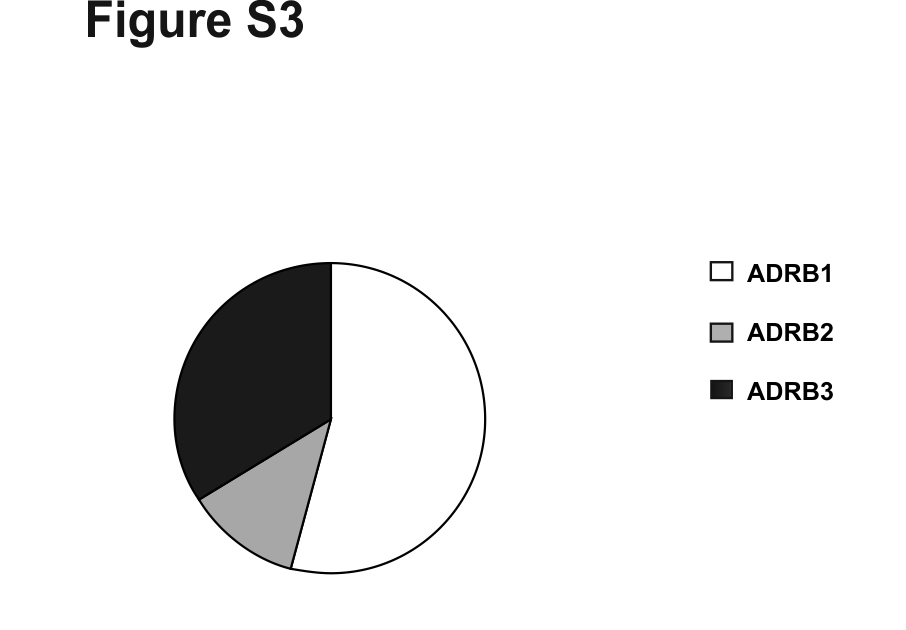

Supplement: S3 Fig — Expression of ADRB1, ADRB2 and ADRB3 mRNA was analyzed by qPCR in differentiated SGBS cells. Data are expressed as percentage of total. ADRB1: Adrenergic receptor, beta 1; ADRB2: Adrenergic receptor, beta 2; ADRB3: Adrenergic receptor, beta 3. (TIF) [file pone.0129644.s003.tif]

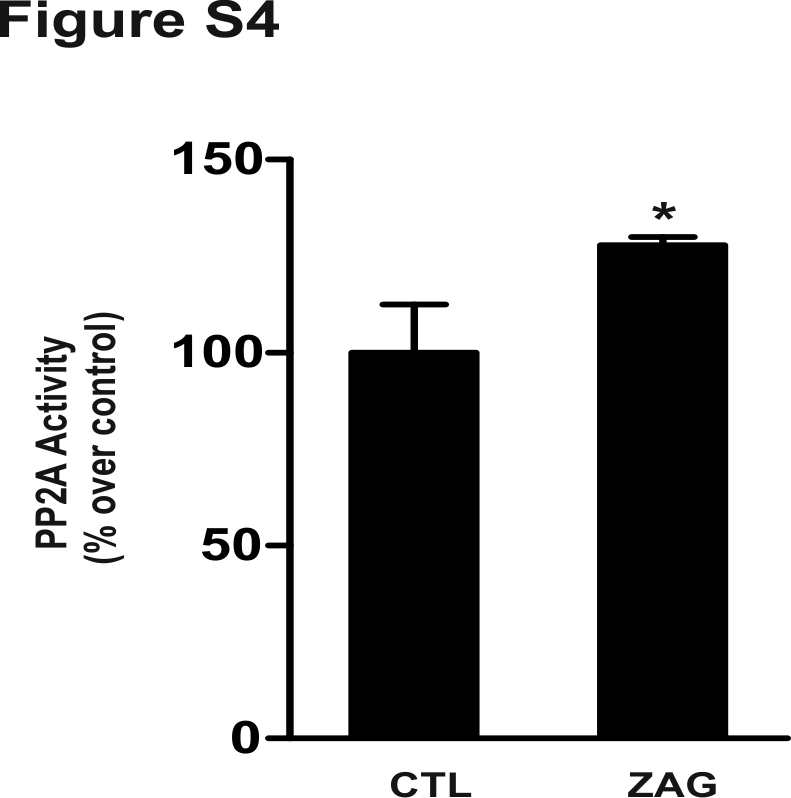

Supplement: S4 Fig — Differentiated LiSa-2 cells were incubated with 25ìg/ml ZAG for 24 hours, and PP2A activity was measured as described in Materials and Methods. Results represent means ± SE of 3–4 independent experiments. Significant differences: *, p<0.01 vs. control. (TIF) [file pone.0129644.s004.tif]
